# Sub-Millisecond Phase Retrieval for Phase-Diversity Wavefront Sensor

**DOI:** 10.3390/s20174877

**Published:** 2020-08-28

**Authors:** Yu Wu, Youming Guo, Hua Bao, Changhui Rao

**Affiliations:** 1 The Key Laboratory on Adaptive Optics, Chinese Academy of Sciences, Chengdu 610209, China; wuyu18@mails.ucas.ac.cn (Y.W.); hbao@ioe.ac.cn (H.B.); chrao@ioe.ac.cn (C.R.); 2 Institute of Optics and Electronics, Chinese Academy of Sciences, Chengdu 610209, China; 3 University of Chinese Academy of Sciences, Beijing 100049, China

**Keywords:** phase diversity, convolutional nerual network, real time

## Abstract

We propose a convolutional neural network (CNN) based method, namely phase diversity convolutional neural network (PD-CNN) for the speed acceleration of phase-diversity wavefront sensing. The PD-CNN has achieved a state-of-the-art result, with the inference speed about 0.5 ms, while fusing the information of the focal and defocused intensity images. When compared to the traditional phase diversity (PD) algorithms, the PD-CNN is a light-weight model without complicated iterative transformation and optimization process. Experiments have been done to demonstrate the accuracy and speed of the proposed approach.

## 1. Introduction

Adaptive optics (AO) is widely used in large astronomical telescopes for turbulence induced wavefront distortion compensation [1]. Wavefront sensing is the key technology of AO, and researchers have done extensive research on wavefront sensing technologies. Traditional wavefront sensing technologies [2,3,4,5] include Shearing Interferometers (SI), Shack-Hartmann Wavefront Sensors (SHWFS), the curvature WFS, etc. SI has high measurement accuracy, but low light energy utilization and complicated optical path. SHWFS is widely used in AO systems, but limited by low spatial resolution due to its pupil segmentation mechanism. When compared to SHWFS and SI, the phase diversity (PD) method proposed in [6] has a simpler optical path and no non-common optical path aberration [7,8]. However, due to high computational complexity, the PD method is mainly applied in the field of post-processing of blurred image and areas with lower real-time requirements [9,10].

Recently, with its rapid development, artificial intelligence has become a very powerful tool in various fields. Machine learning, including deep learning, has also become a hot topic in the field of optics and photonics [11]. As early as 1994, Kendrick et al. [12,13] used neural network technology in the PD method, but there was no consideration of the real-time performance of the algorithm. Georges III et al. [14] proposed a proof-of-concept phase-diversity wavefront sensing and control testbed that displayed 5/1000 wave Root Mean Square accuracy, operated at an estimation rate of 100 Hz. Dolne et al. [10] proposed an approach for real-time wavefront sensing and image enhancement that could process PD images at 50 to 200 Hz. Miyamura et al. [15] also used a neural network to solve the complicated inverse problem of the PD method. Principal component analysis (PCA) is used for the preprocessing of the neural network to compress the information to reduce computation cost. In the last two years, machine learning has been increasingly applied to phase retrieval. Paine et al. [16] used machine learning operating on a point-spread function in order to determine a good initial estimate of wavefront. The convolutional neural network (CNN) outputted a prediction in 0.2 s, while the nonlinear optimization took 16 seconds on average with a desktop computer. Ju et al. [17] proposed a novel phase retrieval mechanism using machine learning that estimated aberration coefficients from Tchebichef moment features. This method is more robust, but still less accurate than traditional iterative phase recovery algorithms. Guo et al. [18] proposed a phase-based sensing approach using machine learning, which can directly estimate the phase map from the point spread functions. With the same accuracy, the stochastic parallel gradient descent algorithm (SPGD) took 448 ms, while the phase-based sensing approach took 11 ms. Nishizak et al. [19] experimentally demonstrated a variety of image-based wavefront sensing architectures that can directly estimate aberration coefficients from a single intensity image by using Xception network [20]. This method still has a large aberration measurement error, and the estimation time was 9.2 ms for a single image. Andersen et al. [21] used InceptionV3 to analyze both a focal image and a slightly defocused image. However, no experimental data were used to demonstrate the effectiveness in practical situations. Ma et al. [22] proposed a novel wavefront compensation method based CNN that only require two intensity images detecting for each distorted wavefront compensation. However, there is also a degree of discrepancy between simulation and experiment. The average prediction time for the CNN after training was 0.16 s. Xin et al. [23] proposed an image-based wavefront sensing approach while using the deep long short-term memory (LSTM), which is applicable to both point source and any extended scenes.

So far, these studies have tended to explore the possibility that deep neural network algorithm can partially or completely replace traditional phase iterative algorithms in terms of accuracy. The calculation time of algorithms ranges from about 10 ms to several seconds due to differences in networks or hardware conditions, which cannot meet the correction speed requirements of modern astronomical adaptive optics systems on the time scale of millisecond or even sub-millisecond [24]. Our work here focuses on both accuracy and the real-time performance of the algorithm.

We propose a novel real-time non-iterative phase-diversity wavefront sensing that successfully establishes the nonlinear mapping between intensity images and the corresponding aberration coefficients by using phase diversity convolutional neural network (PD-CNN). We improve the real-time performance of the algorithm using TensorRT and reduce the aberration measurement error by fusing focal and defocused intensity images. After optimization, the PD-CNN proposed only needs about 0.5 ms for the phase retrieval procedure. Experiments have been done to demonstrate the accuracy and speed of the proposed approach.

## 2. Experimental Setup

**Figure 1:**
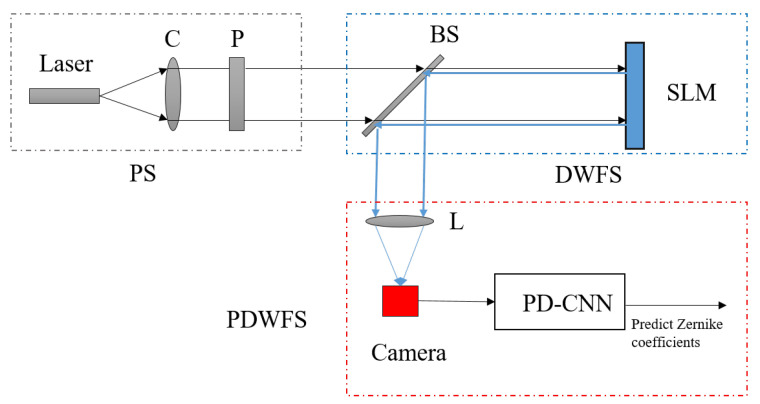
The experimental optical system for generating data sets.

The experimental optical system used to generate the data sets consists of three main parts: a source (S), a distortion wavefront simulator (DWFS) and a phase-diversity wavefront sensor (PDWFS), as shown in Figure 1. The S is composed of a laser (658 nm), a collimator (C), and a linear polarizer plate (P). The DWFS is mainly used to generate aberration and it consists of a beam splitter (BS), and the spatial light modulator (SLM, pixel pitch: 15μm ×15μm, pixel format: 512×512, Model: PCle 8-bit). The PDWFS mainly includes lenses, Camera (Basler acA780-75gm GigE, pixel pitch: 8.3μm ×8.3μm, pixel format: 782×582, Model: 8-bit), and PD-CNN. In the part of S, the P is used to make the polarization direction of light conform to the requirements of the SLM, and the SLM is used to distort wavefront. Finally, the real PSF images are detected by the Camera, which are the inputs of PD-CNN for predicting corresponding Zernike coefficients.

**Figure 2:**
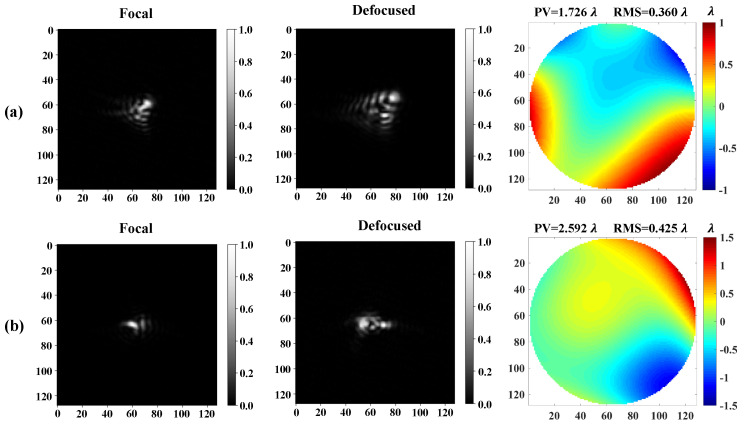
The example of data set. (**a**,**b**) the example of two pairs of the focal and defocused images and their corresponding phase map obtained by the Zernike coefficients. The pixel value of the focal and defocused images have been normalized to 0–1 before inputting to the neural network and the size of images is 128×128. The unit of phase map is λ.

For simulating atmospherically distorted wavefronts, firstly, the independent random Karhunen–Loeve coefficients with the atmospheric conditions $D/r_{0}=10$ can be computed, then be converted to Zernike coefficients according to the Karhunen–Loeve Zernike expansion [25]. Each set of Zernike coefficients can be used to generate the corresponding phase pattern via the Zernike polynomial, which are loaded on the SLM to distort the wavefront. The first and second Zernike coefficients are both set to zero to exclude the tip-tilt. The Camera is displaced at the focal plane of L to detect the focal intensity images. For detecting defocused intensity images, we add an additional defocused aberration whose peak to valley (PV) value is equal to one wavelength. In this paper, there are a total of 6000 pairs of samples in the training data sets, 1000 pairs of samples in the validation data sets, and 3000 pairs of samples in the test data sets. Each pair consists of a group of Zernike coefficients as the label and the corresponding focal and defocused images as the input. Two examples of data sets are shown in Figure 2.

## 3. Method

### 3.1. The Phase Retrieval Approach Using PD-CNN Models

**Figure 3:**
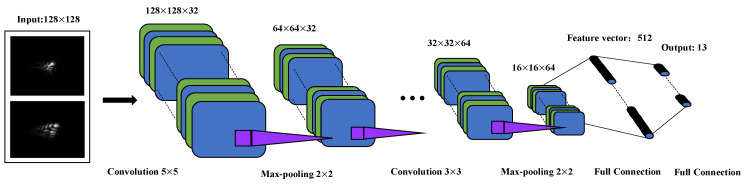
The architecture of the phase diversity-convolutional neural network (PD-CNN) network.

LeNet-5 [26] is a CNN originally used for handwritten digit recognition. In this paper, we have improved the LeNet-5 network for phase retrieval, named PD-CNN, and the architecture of it is shown in Figure 3, including three convolution layers, three max-pooling layers, and two full connection layers. The configuration parameters of it are shown in Table 1. The activation function of all hidden layers are the rectified linear unit (ReLU) function [27]. The images that are acquired by the camera are cropped to 128×128 as inputs. The outputs of the last max pooling are reshaped and sent to the fully connected layers. The last fully connected layer outputs 13 parameters that refer to the predicted Zernike coefficients.

The parameters of the convolutional kernels are updated during network training process to obtain accurate feature information. The pooling layer can compress feature information extracted from the previous layers, removing redundant information, and reducing the complexity of the network. The max-pooling layers are not only used to reduce the computational cost of next layer, but also prevent overfitting [28]. Each node of the fully connected layer is connected to all nodes of the previous layer, which can synthesize the previously extracted features. The cost function used in the PD-CNN is Mean Square Error (MSE), which is used to estimate the degree of inconsistency between the outputs and the target values. In this paper, MSE means the difference between the predicted Zernike coefficients and the target Zernike coefficients. Compared to the deep neural networks (i.e., Xception, InceptionV3), used for phase recovery, the PD-CNN models are smaller with fewer parameters and easier to achieve inference acceleration. Therefore, it has considerable advantages in real-time phase retrieval.

### 3.2. The Inference Acceleration of PD-CNN Model

The application of deep learning has always been a problem in real time, so the inference acceleration for deep learning has also become a hot topic of current research. At present, methods [29,30,31] for inference acceleration include Pruning, Quantification, Distillation, and optimization of network structures. In this paper, we use TensorRT 5.0 to accelerate the inference of the best PD-CNN model saved during the whole training process. There are three steps, importing the Keras model, building an optimized TensorRT engine and performing inference. The core of NVIDIA TensorRT is a C++ library that facilitates high-performance inference on NVIDIA graphics units (GPUs). It focuses specifically on running an already-trained network quickly and efficiently on a GPU for the purpose of generating a result.

## 4. Result

**Figure 4:**
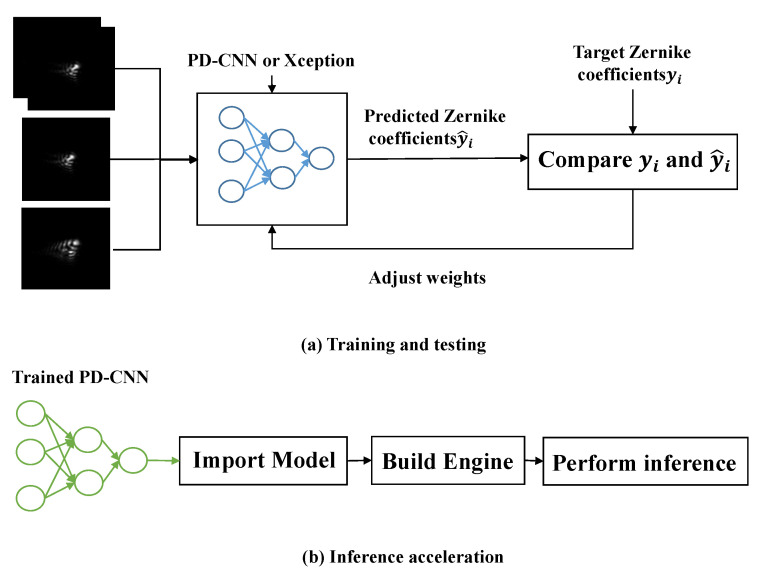
The experiments in this paper. (**a**) the part of training and testing neural network. (**b**) the part of inference acceleration.

The experiment in this paper consists of two parts, training and testing neural networks and inference acceleration, as (a) and (b) shown in Figure 4. The purpose of part (a) is to obtain a trained optimal model for phase retrieval. The purpose of part (b) is to explore the advantages of the trained optimal model in real time. In part (a), the difference of training and testing neural network is that testing does not need to adjust weights. In the process of training, the neural network, the weights are adjusted by comparing predicted Zernike coefficients ${\hat{y}}_{i}$ and target Zernike coefficients $y_{i}$. Once the neural network is well trained, we can save the weights of the network then do the inference. For comparison, we use PD-CNN and Xception to restore wavefront. For each network, three sets of contrast experiments are set as follows, inputting the focal and defocused intensity images separately, and inputting the focal and defocused intensity images at the same time. Finally, we explore inference acceleration of PD-CNN models on embedded platforms Jetson AGX Xavier and 1080Ti. Part (b) shows the workflow of TensorRT5.0 used in this paper. There are three steps, as described in Section 3.2. The input is a trained optimal model of PD-CNN.

### 4.1. The Experimental Results of Training and Testing Neural Networks

Firstly, we set up the PD-CNN network by Keras framework based on Python 3.6.8 to perform regression analysis. The training and testing data sets are generated, as described in Section 2. During the training, we use a learning algorithm, called adaptive moment estimation (Adam), to optimize it with an initial learning ratio of 0.0001, a batch size of 32, and the number of epochs is 100. There are three contrast experiments of PD-CNN, as described in the part (a). The trained optimal models are respectively named Focal model with focal intensity images as inputs, Defocused model with defocuses intensity images as inputs, and PD model with focal and defocused intensity images at the same time as inputs. Secondly, we train the Xception network used in [19] with the same data sets, and the parameters of network are also the same. In addition, the three contrast experiments are also same as PD-CNN. The codes execute on a computer with an Intel Xeno CPU E5-2609 v4 CPU running at 1.7 GHz, with 64 GB of RAM, and an NVIDIA GeForce GTX 1080Ti with 11 GB of RAM.

**Figure 5:**
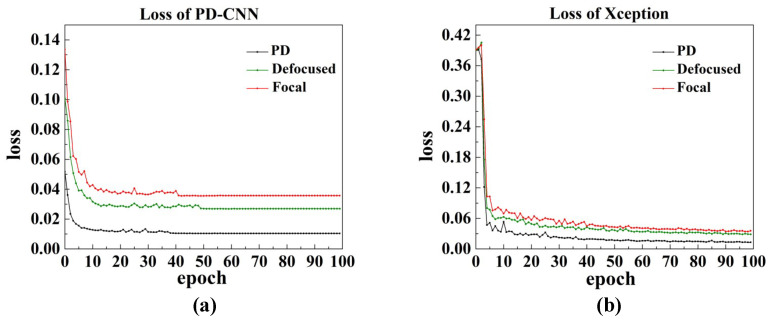
(**a**) The training curves of PD-CNN. (**b**) The training curves of Xception.

**Figure 6:**
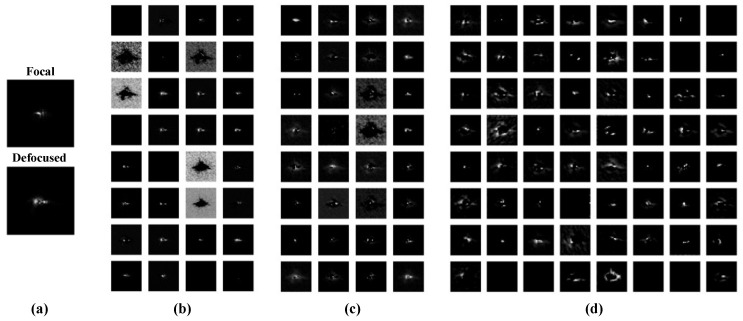
The output feature maps from (**b**) convolution layer 1, which are 32 images of 128×128 pixels, (**c**) convolution layer 2, which are 32 images of 64×64 pixels, and (**d**) convolution layer 3, which are 64 images of 32×32 pixels, when the input a pair of focal and defocused images of test data set shown in (**a**) to the trained PD-CNN.

Figure 5 shows the training process of PD-CNN and Xception network, where the three sets of contrast experiments of them are successfully converged. As the results shown in the Table 2, the loss (MSE) of the three sets of contrast experiments of PD-CNN are 0.0372, 0.0279, and 0.0109, respectively. The PD model has the minimal MSE. Figure 6 displays the feature maps after each convolution layers of one example in a trained PD model of PD-CNN. As shown in Table 3, the average inference time of the three PD-CNN models on 1080Ti is close, about 2.2495 ms, 2.2989 ms, and 2.5591 ms, respectively. The loss (MSE) of the three Xception models are 0.0357, 0.0295, and 0.0139. The results of PD-CNN and Xception both prove the improvement of accuracy by fusing focal and defocused intensity images. Compared with PD-CNN, Xception models need more time for inference and the time are 10.4690 ms, 10.1108 ms, and 10.4690 ms, under the same condition. The inference speeds of the three Xception models are also very close. The accuracies of PD-CNN models and the corresponding Xception models are close, while PD-CNN has an advantage in inference speed.

**Figure 7:**
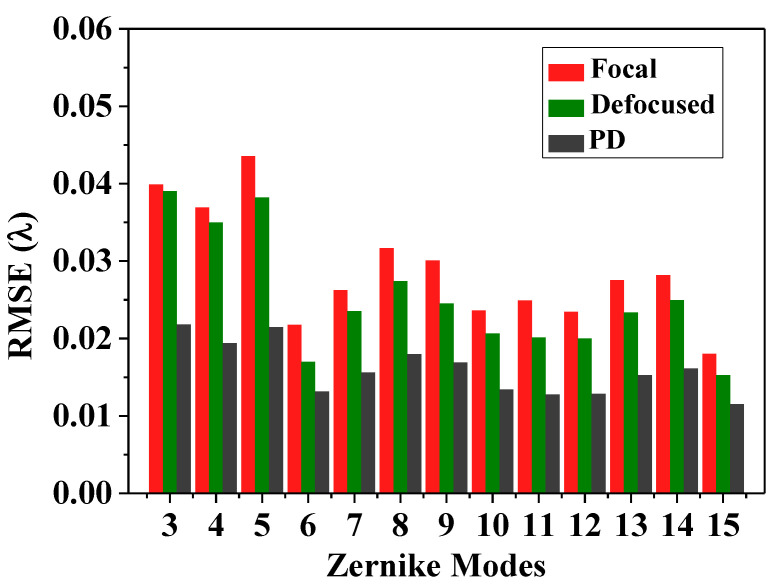
The accuracies (RMSEs:λ) of each Zernike coefficient estimated by PD-CNN across 3000 samples.

**Figure 8:**
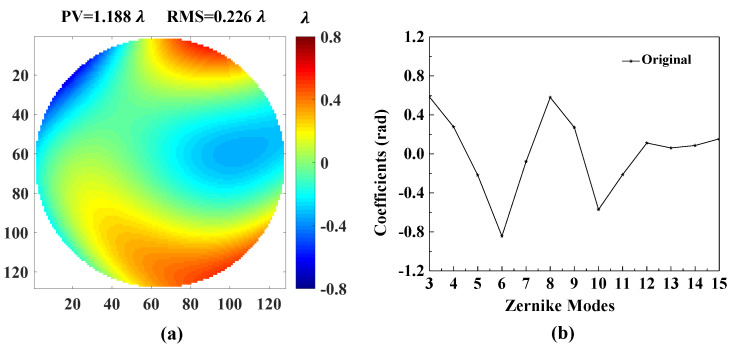
A sample of test data set. (**a**) The original wavefront and (**b**) the original Zernike coefficients. The unit of original wavefront is λ. The unit of original Zernike coefficients is rad.

**Figure 9:**
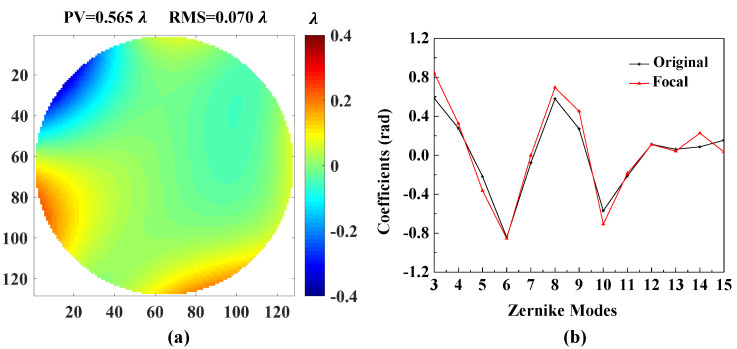
The result of the Focal model of PD-CNN. (**a**) The residual wavefront and (**b**) the comparison of the Figure 8b and the estimated Zernike coefficients. The unit of residual wavefront is λ. The unit of estimated Zernike coefficients is rad.

**Figure 10:**
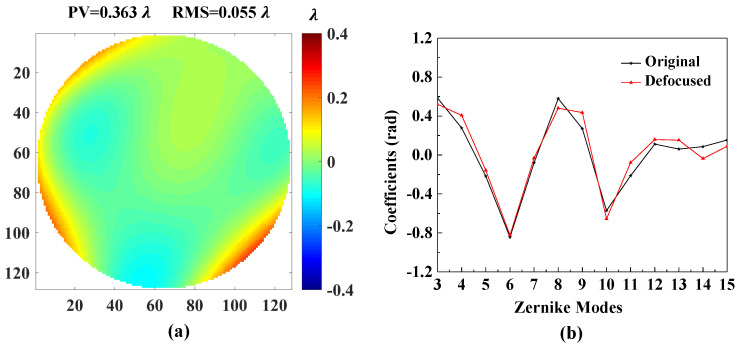
The result of the Defocused model of PD-CNN. (**a**) The residual wavefront and (**b**) the comparison of the Figure 8b and the estimated Zernike coefficients. The unit of residual wavefront is λ. The unit of estimated Zernike coefficients is rad.

**Figure 11:**
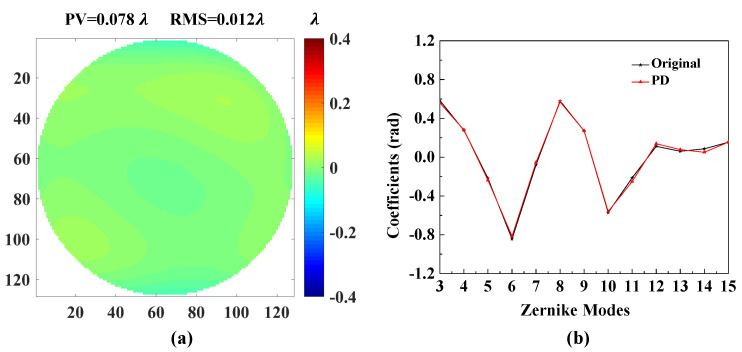
The result of the PD model of PD-CNN. (**a**) The residual wavefront and (**b**) the comparison of the Figure 8b and the estimated Zernike coefficients. The unit of residual wavefro nt is λ. The unit of estimated Zernike coefficients is rad.

**Figure 12:**
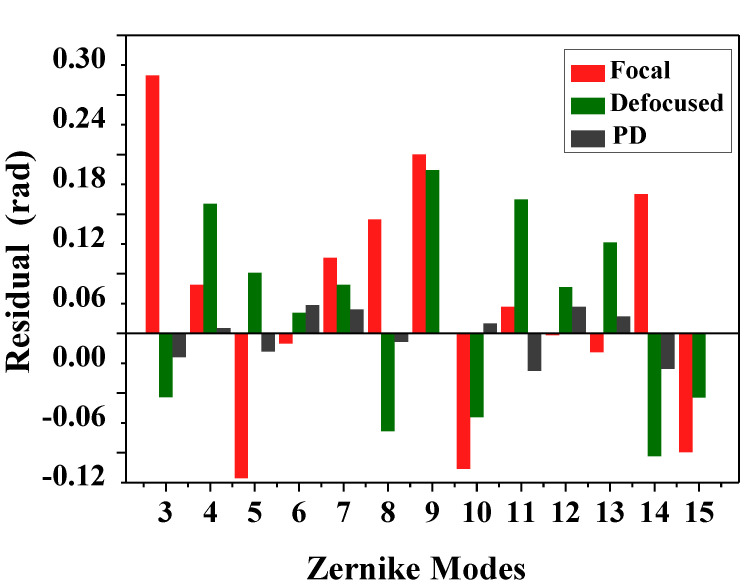
The residual wavefronts of the three models of PD-CNN, when the input the images of the sample shown in Figure 8. The unit of residual wavefront is rad.

Figure 7 shows the accuracies of each Zernike coefficient estimated by PD-CNN. Apparently, the restoration accuracies of each order Zernike coefficients is still consistent with the PD model being optimal, followed by the Defocused model, and the Focal model has the worst conclusion. The results of PD-CNN models in test data set are shown in Table 4. The Original RMSE (Root Mean Square Error) and the standard error of test data set is 0.3398±0.0940λ. The Relative RMSE is equal to the ratio of Estimated RMSE to Original RMSE. The PD model has the smallest Estimated RMSE and the best robustness. Figure 8 shows a sample of test data set. The RMSE and PV of it are 0.026λ and 1.188λ. Figure 9, Figure 10 and Figure 11 show the results of the PD-CNN models with this sample as input. Figure 12 shows the residual wavefront of the three models. The residual wavefront is equal to the estimated wavefront minus the original wavefront. It can be intuitively seen that the PD model has the smallest measurement error.

### 4.2. The Experimental Results of Inference Acceleration

**Figure 13:**
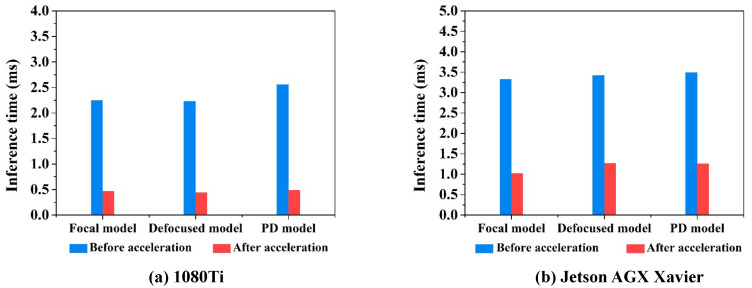
(**a**) The average inference time of PD-CNN models for 3000 samples on 1080Ti. (**b**) The average inference time of PD-CNN models for 3000 samples on Jetson AGX Xavier.

When compared with Xception, the PD-CNN network has an advantage in reference speed, so we further explore inference acceleration of PD-CNN. Firstly, we use TensorRT5.0 to optimize the PD-CNN models by combining layers and optimizing kernel selection for improving latency, throughput, power efficiency, and memory consumption, which are the critical factors that are used to measure the performance of software for inference of trained network. As the inference time of PD-CNN models shown in Table 5, the inference time of Focal model, Defocused model, and PD model on 1080Ti are as follows: 2.2495 ms, 2.2989 ms, and 2.5591 ms. After acceleration with TensorRT5.0, the inference time of them are 0.4678 ms, 0.4406 ms, and 0.4909 ms. As shown in Figure 13a, the inference speeds of the three models are very close, and PD model has the largest acceleration ratio.

In addition, we also explored the inference speed of the three PD-CNN models on the embedded platform, Jetson AGX Xavier of NVIDIA with TensorRT5.0, which can process data at the data source port with limited resource. As the results that are shown in Table 6, the inference time of the three models of PD-CNN on Xavier are as follows: 3.3312 ms, 3.4183 ms, and 3.4854 ms. After acceleration, the inference time of them are 1.0228 ms, 1.2654 ms and 1.2642 ms. As shown in Figure 13b, the inference speeds of the three models are also very close, and the Focal model has the largest acceleration ratio. Although the inference time on the Jetson AGX Xavier platform are larger than 1080Ti, the inference acceleration on the embedded platform has more application value.

Finally, we try a lightweight network ShuffleNet to restore phase, which is optimized for network structures, but the experimental results are not converged. Although we only optimize the model structure and computing resource allocation with TensorRT5.0, and do not reduce the accuracy of the model parameters, the accuracy of the three models are both lost, as shown in Table 7, compared between Table 4 and Table 7, the losses are within acceptable limits. Nevertheless, PD model still has the smallest RMSE and standard error after acceleration.

## 5. Conclusions and Discussion

In this paper, we propose a novel real-time non-iterative phase-diversity wavefront sensing, which successfully establish the nonlinear mapping between intensity images and the corresponding aberration coefficients by using PD-CNN. There is no need for time-consuming iterative transformation or optimization process when compared with conventional phase retrieval approaches. The PD-CNN is a light-weight model and easy to achieve inference acceleration when compared to current phase retrieval using CNNs (i.e., Xception, De-VGG). The optimization of PD-CNN by using TensorRT has two main aspects. One is to analyze the network structure and combine similar calculations to reduce data computation time. The other is to optimize the parameter allocation of NVIDIA GPU resources. After optimization, the inference time of PD-CNN can meet the correction speed requirements of modern astronomical adaptive optics systems on the time scale of millisecond or even sub-millisecond. Experiments have been done to demonstrate the accuracy and speed of the proposed approach.

The experiment in this paper consists of two parts. We use different types of CNNs for experiments, and each CNN has done three sets of contrast experiment, as shown in Section 4.1. Among them, the results of Xception and PD-CNN are the best of inputting the focal and defocused intensity images at the same time. To a certain degree, the accuracy of phase recovery is improved by fusing focal and defocused intensity images. From the perspective of inference acceleration of deep learning algorithms, we explored the application prospects of PD-CNN in real-time wavefront restoration system, as shown in Section 4.2. After the three steps of optimization with TensorRT5.0, the reference time on 1080Ti only needs about 0.5 ms, achieving a state-of-the-art result.

This work presents a simple and effective method to improve the accuracy and real-time performance of phase-diversity wavefront sensing. We accurately recover the first 15 order Zernike coefficients (first and second coefficients are constant at zero). In future work, we will upgrade the experimental system to use 16-bit SLM and camera, and optimize PD-CNN to accurately recover the first 65 Zernike modes. The accuracy loss analysis after acceleration is also the focus of the next research work. In addition, we will explore the CNN based phase-diversity wavefront sensing for extended sources.

## Figures and Tables

**Table 1:** The configuration parameters of the PD-CNN network.

Layer Number	Type	Input Size	Filter Size	Stride	Pad	Number Kernels
1	Input	128×128×2	-	-	-	-
2	Convolution	128×128×2	5×5	1	2	32
3	ReLU	128×128×32	-	-	-	-
4	Pooling	128×128×32	2×2	2	-	-
5	Convolution	64×64×32	3×3	1	1	32
6	ReLU	64×64×32	-	-	-	-
7	Pooling	64×64×32	2×2	2	-	-
8	Convolution	32×32×32	3×3	1	-	64
9	ReLU	32×32×64	-	-	-	-
10	Pooling	32×32×64	2×2	2	-	-
11	Full Connected	16×16×64	-	-	-	-
12	Full Connected	512×1	-	-	-	-

**Table 2:** The Loss (MSE) of test data set in PD-CNN and Xception.

Network	Focal Model	Defocused Model	PD Model
PD-CNN	0.0372	0.0279	0.0109
Xception	0.0357	0.0295	0.0139

**Table 3:** The inference time(ms) of PD-CNN and Xception on 1080Ti.

Network	Focal Model (ms)	Defocused Model (ms)	PD Mode l (ms)
PD-CNN	2.2495	2.2989	2.5591
Xception	10.469	10.1108	10.469

**Table 4:** Summary of accuracies (Root Mean Square Error (RMSE) and standard error: λ of Zernike coefficients estimated by PD-CNN across 3000 samples.

Model	Original RMSE (λ)	Estimated RMSE (λ)	Relative RMSE (λ)
Focal model	0.3398±0940	0.1004±0.0469	0.3016±0.1220
Defocused model	0.3398±0.0940	0.0823±0.0492	0.2434±0.1107
PD model	0.3398±0.0940	0.0529±0.0286	0.1574±0.0721

**Table 5:** The average inference time of PD-CNN models for 3000 samples on 1080Ti.

Model	Before Acceleration (ms)	After Acceleration (ms)	Acceleration Ratio (ms)
Focal model	2.2495	0.4678	4.8091
Defocused model	2.2989	0.4406	5.2178
PD model	2.5591	0.4909	5.2135

**Table 6:** The average inference time of PD-CNN models for 3000 samples on Jetson AGX Xavier.

Model	Before Acceleration (ms)	After Acceleration (ms)	Acceleration Ratio (ms)
Focal model	3.3312	1.0228	3.2569
Defocused model	3.4183	1.2654	2.7014
PD model	3.4854	1.2642	2.7571

**Table 7:** Summary accuracies (RMSE and standard error: λ of Zernike coefficients estimated by PD-CNN after acceleration across 3000 samples.

Model	Original RMSE (λ)	Estimated RMSE (λ)	Relative RMSE (λ)
Focal model	0.3398±0.0940	0.1142±0.1119	0.3455±0.3607
Defocused model	0.3398±0.0940	0.0974±0.1137	0.2910±0.3606
PD model	0.3398±0.0940	0.0696±0.1092	0.2058±0.3247

## Abbreviations

The following abbreviations are used in this manuscript:

MDPI	Multidisciplinary Digital Publishing Institute
DOAJ	Directory of open access journals
TLA	Three letter acronym
LD	linear dichroism
